# Surface EMG in Clinical Assessment and Neurorehabilitation: Barriers Limiting Its Use

**DOI:** 10.3389/fneur.2020.00934

**Published:** 2020-09-02

**Authors:** Isabella Campanini, Catherine Disselhorst-Klug, William Z. Rymer, Roberto Merletti

**Affiliations:** ^1^LAM-Motion Analysis Laboratory, Neuromotor and Rehabilitation Department, San Sebastiano Hospital, Correggio, Azienda USL-IRCCS di Reggio Emilia, Reggio Emilia, Italy; ^2^Department of Rehabilitation & Prevention Engineering, Institute of Applied Medical Engineering, RWTH Aachen University, Aachen, Germany; ^3^Shirley Ryan Ability Lab, Single Motor Unit Laboratory, Chicago, IL, United States; ^4^Laboratory for Engineering of the Neuromuscular System (LISiN), Department of Electronics and Telecommunications, Politecnico di Torino, Turin, Italy

**Keywords:** surface electromyography, sEMG, rehabilitation, clinical applications, motion analysis, education, physiotherapy, movement sciences

## Abstract

This article addresses the potential clinical value of techniques based on surface electromyography (sEMG) in rehabilitation medicine with specific focus on neurorehabilitation. Applications in exercise and sport pathophysiology, in movement analysis, in ergonomics and occupational medicine, and in a number of related fields are also considered. The contrast between the extensive scientific literature in these fields and the limited clinical applications is discussed. The “barriers” between research findings and their application are very broad, and are longstanding, cultural, educational, and technical. Cultural barriers relate to the general acceptance and use of the concept of objective measurement in a clinical setting and its role in promoting Evidence Based Medicine. Wide differences between countries exist in appropriate training in the use of such quantitative measurements in general, and in electrical measurements in particular. These differences are manifest in training programs, in degrees granted, and in academic/research career opportunities. Educational barriers are related to the background in mathematics and physics for rehabilitation clinicians, leading to insufficient basic concepts of signal interpretation, as well as to the lack of a common language with rehabilitation engineers. Technical barriers are being overcome progressively, but progress is still impacted by the lack of user-friendly equipment, insufficient market demand, gadget-like devices, relatively high equipment price and a pervasive lack of interest by manufacturers. Despite the recommendations provided by the 20-year old EU project on “Surface EMG for Non-Invasive Assessment of Muscles (SENIAM),” real international standards are still missing and there is minimal international pressure for developing and applying such standards. The need for change in training and teaching is increasingly felt in the academic world, but is much less perceived in the health delivery system and clinical environments. The rapid technological progress in the fields of sensor and measurement technology (including sEMG), assistive devices, and robotic rehabilitation, has not been driven by clinical demands. Our assertion is that the most important and urgent interventions concern enhanced education, more effective technology transfer, and increased academic opportunities for physiotherapists, occupational therapists, and kinesiologists.

## Introduction

### Quantitative Approaches and Measurements in Neurorehabilitation and Physiotherapy

Prevention of injuries and rehabilitation of movement pathologies are among the branches of clinical practice where the impact of technology is leading to improvement of outcomes and to economic benefits. In times of limited resources, the future perspectives and developments of all rehabilitation-related professions will increasingly depend on the evidence supporting the effectiveness of their preventive and therapeutic interventions. This issue has been discussed in a number of scientific contributions and editorials (1–3).

In the last few decades, impressive developments have taken place in many fields providing powerful quantitative approaches toward instrumentation-based assessments in cardiology (ECG, etc.), neurology (EEG, etc.), and biomechanics (inertial sensors, sEMG). Neuroengineering has made available a wealth of investigational techniques and tools, as well as tutorials and textbooks, for understanding mechanisms, implementing prevention, and measuring performance and results of interventions. The proceedings of the International Conferences on Neurorehabilitation (4–8) provide a view of this progress and tools over the last 7 years. A few examples are the devices for the assessment of force, balance, movement, oxygen consumption, and of course, muscle activity. These tools underwent different degrees of translation to the clinics and to the market. This work focuses on surface EMG (sEMG).

**Figure 1:**
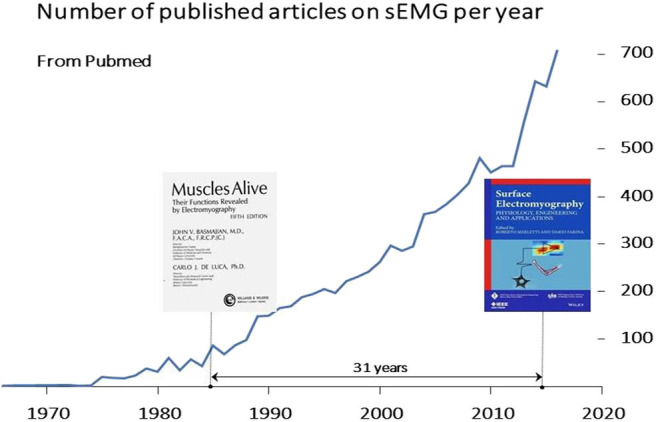
Rate of publication of sEMG articles on international peer-reviewed journals. These articles and more than 20 textbooks (see: https://www.robertomerletti.it/en/emg/material/books/) provide a huge body of knowledge that ranges from technical issues to clinical applications in research labs. A Pubmed search (June 2020, keywords “surface electromyography” OR sEMG) indicated over 5,500 publications (14.8% of the 37,000 publications listed in Pubmed under “neurorehabilitation”). Over 180 review papers are listed by Pubmed in the sEMG field. In most countries, this knowledge is not translated into routine applications for planning treatment, monitoring and assessing outcome in neurorehabilitation.

**Figure 2:**
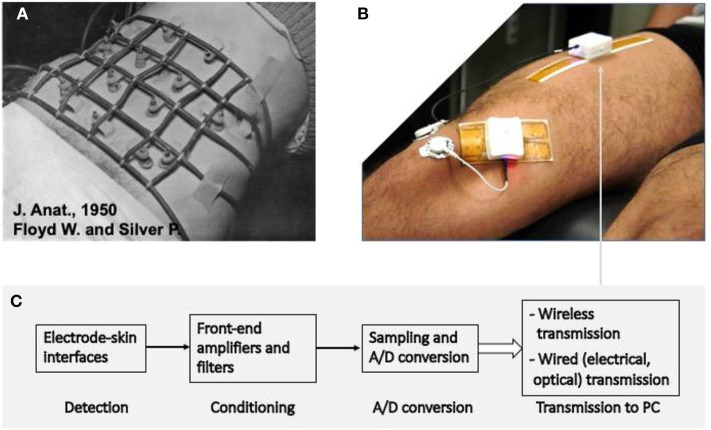
Example of the advances in sEMG detection in the last 70 years. **(A)** The detection system used by Floyd and Silver in 1950 (20) to monitor abdominal muscles. The electronics used for signal conditioning had the size of a suitcase. **(B)** Modern system for detection, condition, A/D conversion, and transmission of signals from sEMG electrode arrays. The white box contains the system described in **(C)** and the rechargeable battery to supply it for a few hours. **(C)** Schematic diagram of the signal detection, conditioning, conversion, and transmission depicted in **(B)**. Two systems, with different detection grids of 32 electrodes each are applied to the rectus femoris and vastus medialis. Up to four such systems can operate simultaneously and provide images of sEMG activity in four locations (21). **(A)** Is reprinted, with permission from Floyd and Silver (20).

As a “muscle activation measuring tool” sEMG has played a growing and important role in neurorehabilitation over four decades (9–19). Figure 1 shows the increase of international peer-reviewed publications in the sEMG field and Figure 2 shows an example of the development of sEMG technology since 1950. Equally striking developments have taken place in related fields of neurophysiology, signal processing and extraction of physiologically relevant features from sEMG over the last 50 years (23–30). Moreover, the number of clinical situations compatible with objective measurements of muscular activity, for planning treatment and for pre- and post-treatment assessment, is large and rapidly increasing, as described in section Surface EMG Applications.

Despite this large body of knowledge, literature, and collected research works, the clinical acceptance of sEMG advances among physiotherapists (PTs), kinesiologists and medical clinicians remains low (31). This is in contrast with the history of ECG in cardiology (32, 33) and EEG in neurology (34). Apparently, the potential benefits of sEMG in assessing treatment appropriateness and in determining cost saving are not fully demonstrated in neuromuscular rehabilitation, primarily because they have not been investigated (22). Research has been focused on academic achievements rather than on clinical applications.

A number of “barriers” exist limiting the widespread application of sEMG techniques in clinical assessment and in neurorehabilitation. Some barriers are cultural, such as the inappropriate comparison with the diagnostic power of needle EMG (35, 36), or are related to the issue of assessing “function” (with scales and observational descriptions) rather than “impairment” (with measurement of physical quantities), or to the wide-spread diffidence/reluctance with respect to objective measurement, instrumentation, and Evidence Based Practice (EBP) (37–40), or the belief that time spent in assessing results is not productive. There is often a lack of a common language with rehabilitation engineers and many therapists lack the technical background to interpret the sEMG outcomes. Some barriers are technical, like difficulties with the application of sEMG, signal processing and information extraction algorithms which do not directly produce clinically relevant information, or the user-unfriendliness of some equipment. Finally, the cost of the devices, the reimbursement procedures, and the time needed to perform a measurement and obtain a clinically useful information have also to be taken into account.

It is the objective of this work to discuss these barriers and the options for reducing them.

### The Role of sEMG for Monitoring Disorders, Planning Treatments, and Assessing Their Effectiveness

Surface EMG can be used in monitoring neuromuscular pathologies, in prevention of work-related disorders and occupational therapy, and in monitoring neuromuscular changes/progress in acute patients (see section Surface EMG Applications). Information on muscle activation during a movement or effort adds to the clinical evaluation and provides a picture of both impairment and functional alteration. As in other branches of medicine, clinical assessment does not always provide the information needed to design a treatment plan. It is crucial to recognize situations in which added value can be expected from instrumental analysis. Some example of questions which can be answered with sEMG, and which have an impact on designing a rehabilitation plan, are presented in section Some Fundamental Questions. Measurement of muscle activation provides information on the motor unit recruitment/derecruitment capability, on fatigue, synergies, co-contractions, etc. as well as evidence of the efficacy of the rehabilitation plan. Section Surface EMG Applications provides examples of applications.

### Physiological and Technological Literacy: The Need for Academic Education and Large-Scale Studies

Physiological and technological literacy is a requirement for medical and health-allied professionals.

The measurement of a physiological quantity (e.g., localized myoelectric manifestations of fatigue) is useless if the recipient does not know its meaning, how to use the information contained in the result and how reliable the measurement is. An ECG does not convey much information to a person knowing little about cardiac electrophysiology. Similarly, information about muscle fiber conduction velocity, sEMG patterns, and amplitude or frequency spectrum, etc. do not inform a person knowing little about muscle electrophysiology and basic signal analysis. The insufficient competence of instrument operators in performing and interpreting such measurements leads to the predictable conclusion that measurements do not contain clinically useful information.

In many countries, the lack of this literacy in physiology/technology leads to the education of physiotherapy and occupational therapy graduates, and future teachers, who serve primarily as professional operators with empirical knowledge mainly (41). This is confirmed, in many countries, by the lack of scientific publications by these graduates (42). In addition, in many countries, the unavailability of PhD degrees in physiotherapy or occupational therapy precludes the evolution of a full academic career and the training of qualified teachers and researchers, perpetuating the situation in its current state. One consequence is that scientists able to conduct large, badly needed, multi-center studies to document the validity of the information obtained from sEMG, do not yet exist. The absence of qualified operators results in an abundance of scientific papers focused on muscle electrophysiology and sEMG technology, in contrast with the lack of studies on the clinical applications of sEMG and on the clinical testing of usability and effectiveness of this technology.

### Bioelectric Signals: Basic ECG, EEG, and EMG. General Considerations and History

The most important bioelectric signals are generated by the heart (ECG), the brain (EEG), and the muscles (EMG). The technologies for collecting, reading and interpreting these signals were developed 50–60 years ago and reported in many review papers and books (9, 32–34, 43). ECG and EEG are widely accepted for clinical monitoring of heart and brain functions. They are an important part of the training of cardiologists, neurologists and of the associated health operators and technicians. International standards for the detection and interpretation of these signals have been defined decades ago. In contrast, despite the huge amount of international literature from research labs, sEMG suffers from a wide gap between research and clinical applications. This gap is markedly greater in Mediterranean countries than in North-European countries, USA, Canada, and Australia.

Surface EMG is more than a century old. It started with the pioneer work of Piper, Kugelberg, and Denny-Brown (44–46) and progressed in the second half of last century with the fundamental research of Basmajian, Lindstrom, Gydikov, De Luca (9, 23, 24, 43, 47) and the clinical efforts of Kasman, Cram, Kumar, and many others, leading to the systematization of knowledge in sEMG textbooks (10, 11, 18, 19, 48–52). Some of these books are open-access and free for download.

The sEMG signal is the algebraic sum of the motor unit action potentials (MUAP) generated by the active motor units (MU) and detected over the skin. Like any other signal, sEMG provides quantitative information concerning wave-shape, amplitude, power spectral density, etc. Using such information or those derived by visual observation is a clinical choice/decision.

Traditionally, this signal is detected between two electrodes aligned in the direction of the fibers (bipolar or single differential electrode montage). This “conventional” bipolar sEMG provides ready answers to many important questions in rehabilitation. It is simple to apply on multiple muscles, even by non-technical users, and provides reliable information on the general activation of a muscle or muscle groups and on temporal events of muscular activation (53). Conventional bipolar sEMG is applicable in almost all clinically relevant situations, such as in dynamic movements, in isometric contractions, and in patients with severe movement disorders, adults and children.

Advanced sEMG technologies provide a much larger amount of physiological information than the simple bipolar technique. For example, the use of surface electrode arrays, enables the detection of a so called sEMG “image” that is evolving in time like a movie on the skin [see examples in (52)]. This time-varying electrical image provides indirect information on muscle force, on motor unit (MU) recruitment and de-recruitment strategies and discharge rates, on muscle length, on the location of the innervation zones (IZs), myoelectric manifestations of muscle fatigue, and many other phenomena of interest in neurophysiology, neuropathology, neurorehabilitation, ergonomics, aging, sport, and space medicine (14, 54–60). This technique has been applied in research labs and has been ready for large clinical studies for a few decades. A series of open-access tutorials on this topic is being published in the Journal of Electromyography and Kinesiology (61, 62).

The current applications of sEMG mostly concern physiological investigations, monitoring of neurological disorders, planning of treatments, assessment of interventions and control of prostheses and robots (section Surface EMG Applications). For reasons of space, only a few references are provided here for each area of application. A much more extensive description of applications is provided in chapters 10–20 of Merletti and Farina (18).

## Surface EMG Applications

### General Fields of sEMG Application

Like other bioelectric signals, sEMG provides a fundamental added value to the assessment of the organ generating it. The information about muscle activation has different forms (amplitude, timing, morphology, spectral features, muscle fiber conduction velocity, displacement of innervation zone, contributing synergies, muscle coordination, control strategy, etc.) and is relevant in many fields ranging from orthopedics and neurorehabilitation, to movement analysis in exercise and sport, from aging to gnathology, from obstetrics to occupational and space medicine (14, 54–60). Each of the specific assessment methods and techniques listed in Table 1 and section Overview of sEMG Applications is transversal to most of these medical applications and rehabilitation fields.

**Table 1:** Applications of sEMG.

	**Physiology and basic studies**	**Neurological rehabilitation**	**Orthopedic rehabilitation**	**Gynecological rehabilitation and obstetrics**	**Prosthesis and assistive devices**	**Ergonomics**	**Sport, aging and space medicine**	**Orthodontics and gnathology**
Muscle coordination and activation intervals	x	x	x			x	x	
Primitive synergies	x	x	x					
Spasticity	x	x	x		x			
Muscle over activity		x	x		x	x	x	x
Causes of acquired deformities		x	x		x			
Muscle force estimation	x	x	x	x		x	x	x
Postural control	x	x	x			x	x	
Muscle fatigue estimation	x	x	x		x	x	x	x
Pain	x		x		x	x		
Muscle activity localization	x			x	x	x	x	x
Localization of innervation zones	x			x				
Electrically elicited muscle contractions	x	x	x		x			
Cramps	x	x				x	x	

*Rows list topics, methods, and assessment techniques. Columns list large area of medicine in which such techniques are applicable or are applied*.

Most of the available literature on sEMG concerns methodological issues and proof of concepts, carried out mostly on healthy subjects. Clinical works on large patient groups are few, as well as case studies and case-series on small samples. This does not mean that the developed techniques have no clinical applications or do not answer clinical questions. Rather, it means that there is a huge gap in translating techniques to the clinical environment (see section Barriers to Widespread Clinical Use of sEMG in Neurorehabilitation). Some articles question the diagnostic and therapeutic value of sEMG (35, 36, 63). Conversely, other studies, indicate the use of sEMG as essential in the decision making of functional surgery and in the assessment of spasticity (22, 52, 64–68).

The availability of evidence to support rehabilitation has been a long-standing challenge. The complexity of designing clinical trials to assess the efficacy of rehabilitative treatments has become a topic in the current literature (69). Since sEMG is not a treatment itself, but an instrumental assessment tool that adds to clinical evaluations, specific studies should be designed to assess its impact on (1) the variation in the choice of the rehabilitative pathway and (2) the incremental efficacy of this variation on functional outcomes and on cost/efficacy indicators. A few efforts in this direction can be found in the literature for instrumental gait analysis (22, 70–73). Similar specific studies on sEMG are needed. To address this gap, research teams should include sEMG experts, clinical rehabilitation professionals, rehabilitation engineers, experts in research methodology, and in Health Technology Assessment.

The sEMG signal is affected by a large number of factors reflecting the pathophysiology of the muscle and of its control strategy. As such, it provides a window into the muscle, the peripheral (PNS), and central nervous system (CNS). These factors range from alteration of the MUAP propagation along the motor unit fibers to the control of force by recruitment/derecruitment and firing rate of the individual motor units. Unraveling the large amount of information contained in the signal is a major technological challenge and an important field of current engineering, physiological and clinical research (26, 27, 50, 61, 62). The use of large wireless electrode arrays is today possible and relatively simple (Figure 2B) so that a fast progress is expected for the next decade. The clinical users of these developments should take a primary role by participating to such progress and orienting it; their education should account for the research instruments of today that will be clinical tools tomorrow (62).

### Overview of sEMG Applications

#### Applications in Physiology and Basic Clinical Studies

Basic and clinical neurophysiology are fields in which sEMG has been extensively applied (12, 17, 74–77). The applications listed below are focused on the pathophysiology of muscles whose knowledge is a pre-requisite for planning clinical interventions and solving clinical problems in the neurorehabilitation field.

#### Muscle Coordination

It was demonstrated early on that sEMG is suitable for the detection of co-activation of agonist and antagonist muscles, whereby physiological activation patterns could be distinguished from pathological ones (78–80). The clinical relevance of muscular coordination became stronger with the improvement of sEMG techniques (81) and should now be an integral part of any biomechanical analysis of movement (82, 83). The most common use of sEMG to assess muscular coordination is in clinical gait analysis. Here it can be used either in functional diagnosis or in the monitoring of therapeutic outcomes (84, 85). The most prominent fields of application are for neurological impairments like cerebral palsy (CP) and stroke (86), orthopedic impairments, such as back pain (87), anterior cruciate ligament (ACL) injuries (88), and degenerative joint disease (89). However, the interpretation of sEMG signals with respect to muscular coordination requires some caution (90). For this reason, different signal processing methods have been developed in the past to support the interpretation of sEMG signals (91). Currently, the extraction of sEMG primitive synergies is widely used (92, 93). The most recent approaches to categorization take into account biomechanical factors, on which the sEMG signal depends, when determining the physiological or pathological muscular coordination pattern (89, 94).

#### Extraction of Primitive Synergies

Muscle activation patterns, represented by the sEMG envelopes of a few muscles, can be decomposed into a limited number of “basic” functions or patterns, called “synergies” or “primitives.” These primitive patterns can be combined, with different individual weights, and result in the apparent modular organization of multi-muscle activities across different motor tasks. It has been proposed that the nervous system simplifies muscle control through such modularity, using these basic synergies (primitives) to activate muscles in groups. This discovery has had a huge impact on the analysis of motor control and neurorehabilitation since it implies that the CNS generates forces and movements by optimizing the control strategy of either individual muscles or (more likely) muscle synergies (95–101). Research, largely based on sEMG, is focusing on the alterations of these synergies in stroke and other pathologies.

#### sEMG-Based Muscle Force Estimation

**Figure 3:**
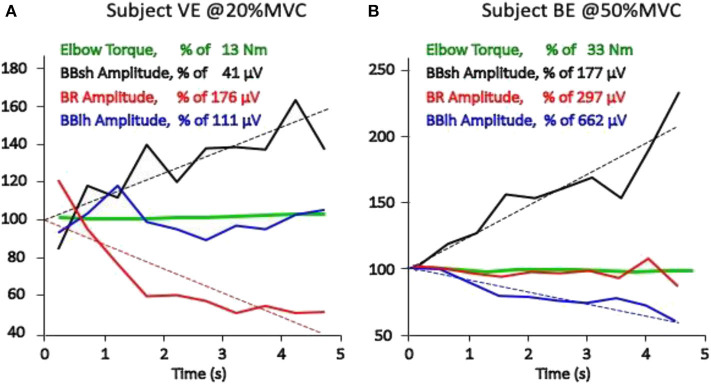
Torque at the elbow and average rectified value (ARV), estimated on epochs of 0.5 s, of the sEMG obtained from three pairs of electrodes placed between the IZ and the tendon endings of the long and short head of the biceps brachii (BBlh, BBsh) and of the brachioradialis (BR) of two healthy subjects (**A** and **B**). All values are expressed as percent of the initial value, which is defined here as the intercept of the linear regression of the experimental values (dashed lines). Results from two 5-s isometric constant torque contractions performed at 20% MVC and at 50% MVC are presented. A progressively changing load sharing among the three muscles is evident and different in the two subjects (**A** and **B**). Different conclusions would have been reached depending on which single muscle had been monitored (unpublished data).

The net torque at a joint is usually produced by a number of muscles, ligaments and other passive structures, and by external forces (e.g., gravity, closed-kinetic-chain forces, orthosis-produced forces). The force contributed by each individual muscle may fluctuate (as does the sEMG amplitude of the muscle) while the total measured torque may remain constant. The estimation of force sharing among synergic muscles by means of sEMG has been reviewed by Perry 30 years ago (102), and more recently, by many other investigators (103–105) but is not yet satisfactorily solved. It is clinically important to realize that one sEMG channel reflects the activity of one (or part of one, or few) superficial muscles while others (including often non-monitored antagonists) may also contribute to the measured torque at the joint. For this reason, care must be taken in associating changes of sEMG amplitude of one muscle to changes of global torque at a joint. At this time, it is rarely possible to acquire the sEMG signal from all muscles acting on a joint. Figure 3 shows two cases of changing sEMG amplitude in three muscles acting on the elbow during two isometric constant force contractions of the elbow flexors. Brachialis and triceps brachii muscles were not monitored. Although the mechanical contribution of each monitored muscle cannot be estimated, the sEMG amplitude trends suggest that the three contributions are changing in time while the total (measured) torque remains constant. Information of this type should be exploited in sport and rehabilitation medicine to teach or modify the muscle activation patterns.

#### Myoelectric Manifestations of Muscle Fatigue

The term “muscle fatigue” has many definitions mostly associated with measurements performed during an isometric constant force contraction which is a common and important “bench-test” condition. One definition considers mechanical fatigue as the inability to sustain a given contraction level and is associated with the endurance time (in isometric constant force contractions) or to the inability to perform a task. Another definition refers to “myoelectric manifestations of muscle fatigue” and considers fatigue as the set of changes affecting sEMG features from the very beginning of the contraction. Its main indicator is muscle fiber conduction velocity (CV) which decreases more or less rapidly depending on the level of contraction and is usually measured during isometric constant force contraction. Both types of “fatigue” depend on blood flow and on the stability of the recruited MU pool. Blood flow is blocked, and all MUs are recruited, at contraction levels above about 50% MVC in most muscles (18, 25, 106–110). In this condition many confounding factors are removed (or are constant) so that an acceptable “bench-test” condition is obtained. The reduction of muscle fiber CV causes a compression of the power spectrum of the sEMG toward the lower frequencies and a decrement of the mean and median spectral frequencies that are generally considered as fatigue indicators but that are affected by many additional confounding factors. Measurements of the myoelectric manifestations of muscle fatigue in intermittent or dynamic contractions are very questionable because of many confounding factors (variable blood flow, variable pool of active motor units, etc.) and require considerable competence and caution in defining the specific measurement protocol and the measurement modalities (30, 77, 111).

#### Muscle Activity Localization

**Figure 4:**
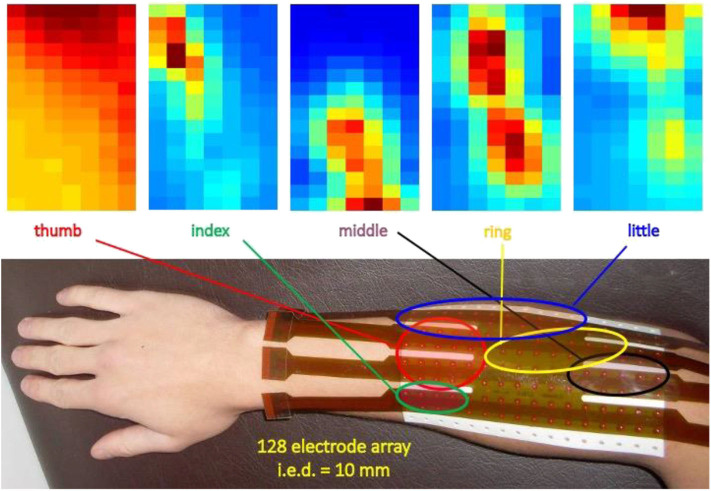
Example of application of a 16 × 8 grid on the dorsal side of the forearm to identify/monitor the regions of activity of the finger extensors. The colors represent the intensity (RMS value) of the longitudinal differential signals (15 × 8 channels). Dark red = strong signal, dark blue = no signal. Interelectrode distance: 10 mm (unpublished data).

The first use of electrode arrays was described by Gydikov in 1972 (23, 24, 52, 112). Identification of innervation zones using electrode arrays was reported by Masuda et al. in 1985 (113, 114) while the technique of “high density” surface EMG (HDsEMG) was developed 10–15 years later (76, 115–119). The technique is also referred to as sEMG imaging and is used to identify active muscles, the geometry of MUs (e.g., fiber length and orientation), and their innervation zone (IZ). Deep muscles (or deep MUs of a superficial muscle) produce force but their sEMG contributions may be near or below the noise level. Techniques to detect such contributions using HDsEMG are being investigated to obtain a sort of “electromyographic tomography” (120, 121). Figure 4 shows a large grid (128 contacts, 10 mm apart) displaying the regions of activity of the extensors of the fingers of the right hand. Similar maps may be obtained for other muscles or muscle groups, such as the erector spinae, the trapezius, etc. Biofeedback applications, for correcting muscle involvement while performing a task, are potentially valuable.

#### Location of Muscle Innervation Zones

A textbook is available with the location of the IZs of 43 muscles (19). Knowledge of the location of the IZs of a muscle is clinically important for (a) proper positioning of a single electrode pair between the IZ and tendon junctions, (b) targeted injection of botulinum toxin (58, 122), and (c) programming surgery in a way that would avoid damage to muscle innervation. The latter application is particularly important for reducing the risk of anal sphincter partial denervation resulting from episiotomy (18, 51, 123–125).

#### sEMG of Electrically Stimulated Muscles

Neuromuscular electrical stimulation involves the application of electrical stimuli to a nerve, or to the motor point of a superficial skeletal muscle, with the objective of inducing and controlling muscle contractions. The stimulus strength (either current or voltage or pulse width) determines the number of recruited motor units whereas the stimulus frequency determines their synchronized discharge rate. Since all the recruited MUs are activated synchronously, effectively as a single large MU, the sEMG signal is deterministic rather than stochastic and is referred to as M-wave or Compound Motor Action Potential (CMAP). Confounding factors and the effects of variability of CNS control, present in voluntary contractions, are eliminated and myoelectric manifestations of muscle fatigue are easy to measure. The technique provides a powerful bench-test for the quantitative investigation of a muscle's electrical and mechanical properties (126–128). Finally, functional electrical stimulation (FES) devices may be triggered or controlled by residual sEMG activity of partially paralyzed limb muscles (129).

### Applications in Neurological Rehabilitation

#### Support for the Assessment and Treatment of Muscle Spasticity and Overactivity

The most common definition of spasticity goes back to Lance, according to whom spasticity is “…a motor disorder, characterized by a velocity-dependent increase in tonic stretch reflexes (muscle tone) with exaggerated tendon jerks, resulting from hyper-excitability of the stretch reflex as one component of the upper motor neuron syndrome” and does “not include impaired voluntary movement and an abnormal posture” (130). Beside this definition, spasticity is a term that is firmly but not consistently used in the clinical environment and among pathologies (131). A brief history of the term, of its use and ongoing evolution can be found in the paper from Baude et al. (132). Not only is a generally accepted definition of spasticity lacking, but there is also a lack and a need of objective methods for assessing the level of spasticity (133, 134).

**Figure 5:**
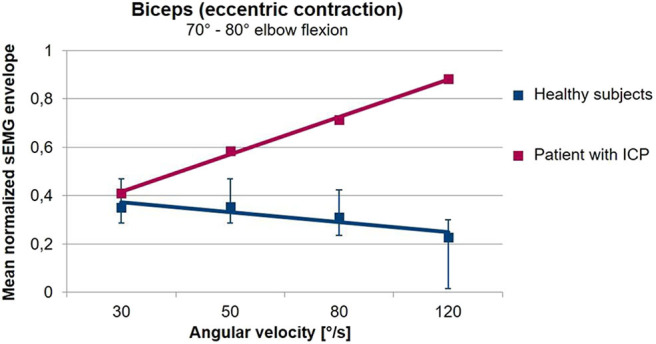
Averaged sEMG envelopes of the Biceps Brachii as a function of the movement velocity during freely performed elbow extension movements. Values are normalized with respect to the 75% of the maximal value of the envelope. The sEMG when the elbow passes an interval from 80 to 70° flexion angle is analyzed. In healthy volunteers (blue), the biceps sEMG envelope decreases with increasing angular velocity. In contrast, in the patient with a spastic movement disorder (red) shown here, muscular activation increases with angular velocity. The gradient of the sEMG envelope–movement velocity relationship is thus a measure for the presence of spasticity during freely performed movements (unpublished data).

Many different studies have shown that sEMG can quantify alterations associated with spasticity, for example through the extraction of sEMG primitive synergies (93). However, these approaches do not distinguish between spasticity, on the one hand, and dystonia, rigidity or voluntary activation, on the other hand. According to the definition of Lance objective assessment of spasticity should be based on the investigation of the tonic stretch reflex. Several studies carried out in the last decades have consequently used sEMG to investigate the muscle response to stretch in the presence of spasticity (135–138). These studies have shown that sEMG provides the easiest and most reliable way of determining the stretch reflex threshold (133). Quantitative assessment of spasticity (139–141) as well as monitoring of treatment are possible (142) by combining sEMG with biomechanical techniques, measuring stretch velocity and torque. More recent approaches use the increased tonic stretch reflex to quantify the occurrence of spasticity during freely performed movements (143–145). Figure 5 shows that in the presence of spasticity, a freely performed extension movement of the elbow leads to an increasing muscular activation with increasing movement velocity. This is in contrast to healthy subjects who use a lower muscular activation when the movement is performed with higher speeds.

#### Support to the Identification of the Causes of Acquired Deformities and Treatment Selection

**Figure 6:**
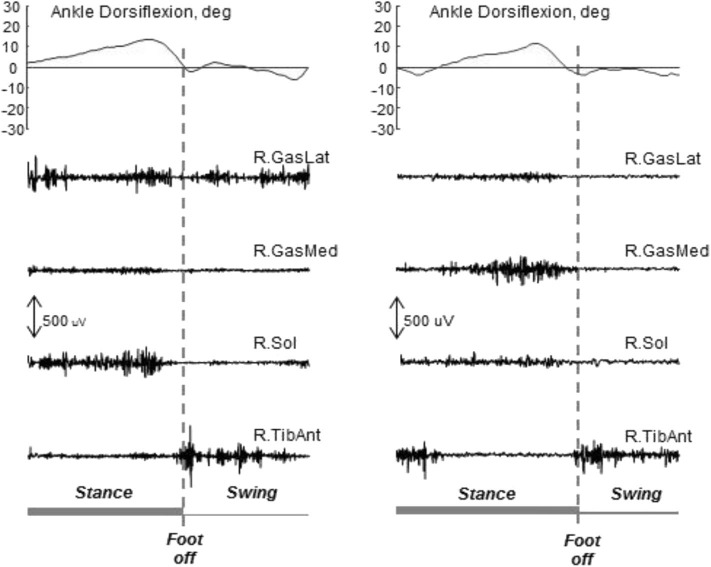
Ankle kinematics and sEMG data from two stroke patients with equinus foot during the swing phase of gait. The same kinematics can be observed, with different underlying mechanisms. In both cases the activity of the tibialis anterior is present at foot off as required to lift the forefoot. On the left, the activity of the gastrocnemius lateralis during the swing phase hinders dorsiflexion. On the right, there is no activity of the triceps surae during the swing phase, and the lack of dorsiflexion is due to the triceps stiffness only. In this situation, sEMG is needed to support the decision-making concerning intervention. Unpublished data acquired in a research project approved by the local Ethics Committee (2017/0123710).

Following lesions to the CNS, such as stroke, traumatic brain injury, etc., patients can develop acquired deformities at the lower limb that impair or inhibit walking. These deformities, often termed contractures, are due to a combination of paresis, muscle overactivity, spasticity, along with mechanical barriers, including muscle shortening, increased muscle stiffness and viscosity, and retractions. Other phenomena (e.g., overactivity, spasticity, lack of recruitment) have to be assessed in dynamic conditions, because they may not be detectable during the bedside evaluation, and may be present only during walking, or vice versa (22, 67, 132). The direct assessment of muscle activity with sEMG and indwelling fine-wire EMG for deep muscles allows discriminating between active and passive causes, thus supporting the selection of treatments tailored for each patient (22, 67, 73, 85, 132). For example, in the assessment of the equinovarus foot deformity in stroke survivors, sEMG of the plantar flexors reveals which muscles are overactive during walking (146). This observation supports the clinical decision-making in choosing among focal muscle blockages, non-pharmacological treatments (147), and neuro-orthopedic or functional surgery. It is worth noting that, in stroke patients the triceps surae muscles can be completely silent during swing with equinus (73). Figure 6 presents data from two stroke patients during walking. Both patients have an equinus foot deformity (i.e., limited dorsiflexion) with the same kinematics. They look equal, based on the visual observation of their gait. Yet, on further analysis of sEMG data, the two equinus deformities have completely different causes, and these are outlined by the sEMG traces (and by sEMG only).

Next, the recruitment of dorsiflexor muscles during walking, regardless of their voluntary activation during tests at the besides, is useful to further tune the surgical plan (e.g., split and transfer of the tibialis anterior tendon) (66, 148). Similarly, sEMG of the quadriceps muscle during walking can be used to support the clinical assessment for selecting the best treatment for stiff-knee gait (22, 65). In stroke survivors, surface EMG can also have a fundamental role in the planning of functional surgery of the upper limb, to support the surgeon's decision about which muscle to lengthen and which muscle insertion to transfer (64). Surface EMG has been used to assess motor function and to support clinical planning of surgical correction of foot deformities in CP children (149). Instrumental gait analysis and sEMG are considered among the fundamental sources of information to drive treatment selection (150).

Surface EMG-derived indices are also used as outcome measures to evaluate the responsiveness to treatments (151). In clinical practice, real-time sEMG can be used by physiotherapists (a) to control if the movement requested to the patient is performed by the proper target muscle(s) or by means of compensatory mechanisms, (b) as a direct measurement of variations consequent to mobilization, verticalization, trunk fixation, in acute neurological patients, (c) to assess the effect of different orthoses on muscle activation, which can vary toward or away from the normal pattern (152).

In conclusion, in patients with either acquired CNS lesions (e.g., stroke, traumatic brain injury, spinal cord injuries) or degenerative diseases (e.g., multiple sclerosis), sEMG can be used (1) to better understand the underlying mechanisms of gait deterioration, especially where multifactorial causes coexists, (2) to support the clinical decision making and/or the rehabilitative pathway, and (3) as a marker of disease progression or intervention effectiveness (153). The same considerations are applicable to many other subsections of the neurorehabilitation field, to exercise physiology, occupational, and sport medicine.

#### Postural Control

Based on the pioneering work of Joseph and Nightingale (154), we now know that many muscles have a postural role that has been investigated by means of sEMG. These muscles oppose the pull of gravity, react to perturbations and allow us to stand, sit, or maintain a desired posture. They achieve this objective by controlling the stiffness of joints (mainly ankle) and by compensating for gravitational forces in either a continuous or an intermittent way. The standing human body is an intrinsically unstable inverted pendulum that requires continuous micro-adjustments to keep the center of mass and the center of pressure within the space defined by the feet.

This mechanism is altered by age and many pathologies and sEMG provides means to monitor such alterations. The role of sEMG is of paramount importance in helping investigators understand how neural regulation contributes to the prevention of falling, by studying the control of posture and the responses to postural perturbations in healthy and pathological subject (155–160).

#### Applications in Orthopedic Rehabilitation

The majority of rehabilitative treatments delivered by physiotherapists are related to orthopedic pathologies. In this field, the available sEMG-based indices assessing muscle activation, symmetry and localized fatigue can be used to support the selection of the therapeutic exercises and to monitor their effectiveness over time (161). In patients with low back pain (LBP), sEMG has been used as a tool for functional diagnosis and to assess the effectiveness of treatment (162, 163) and manipulation (164). Two systematic reviews are available on this topic (164, 165), which concluded that sEMG-based parameters of amplitude and localized fatigue are useful tools to monitor the effect of different interventions delivered to relieve LBP.

At the shoulder level, alterations in neuromuscular control of the scapular muscles has been proven in subjects with subacromial pain syndrome based on sEMG data (166). The effect on muscle activation of myofascial treatment techniques, such as dry needling, has been described in women with trapezius myalgia (167). Neck muscle dysfunction has been quantitively analyzed in patients with cervical spine pain (168, 169). It has to be stressed that the acquisition of sEMG data from the shoulder and neck muscles requires specific training and adequate instrumentation.

Lower limb orthopedic pathologies, related to either sport activity or injuries, have been widely investigated with sEMG-based techniques. For example, sEMG has been used to compare the activation of gluteal muscles between healthy and injured runners, to quantify thigh muscle imbalance in subjects with patellofemoral pain (170–172), and to investigate the causes of Achilles tendinopathy (173). Moreover, sEMG can be used to evaluate residual muscle function and abnormalities in patients who underwent a total hip or knee replacement and to tailor the rehabilitation programs (174). Considerable literature on this topic is available, inclusive of reviews and meta-analyses (171, 173, 175).

#### Applications in the Control of Prosthetic and Assistive Devices

Surface EMG detected from the residual muscles of an amputee has been used for controlling the motors of arm/hand prostheses (myoelectric prostheses) for five decades. This technique is limited to 2–3 basic commands and movements, is not intuitive and requires that the subject learns to associate a specific muscle contraction to the desired output. Recently the technique based on “Targeted Muscle Reinnervation (TMR)” has been tested with success in subjects with amputation at the shoulder level. The residual nerves from the amputated limb are surgically transferred to other, previously denervated, muscles that are not used by the subject. Reinnervation of these muscles takes place within 3–6 months. For example, branches of the median, radial, musculocutaneous, and ulnar nerves may be grafted to specific regions of the serratus or pectoralis muscles. Once reinnervated, these muscles act as biological amplifiers of the neural commands meant for the missing muscles and their sEMG can be detected with electrode arrays and decoded, by pattern recognition processes, for the control of the motors of the prosthesis. The command is therefore intuitive, that is the amputee attempts to move the missing arm and the mechanical arm moves as “desired.” While some problems need to be solved, this technique is highly promising for specific amputees (176–178).

### Applications in Pelvic Floor, Obstetrics, and Gynecologic Rehabilitation

#### Functional Assessment of Pelvic Floor Muscles

Pregnancy and high-impact sport activity are considered as risk factors for pelvic floor dysfunctions, including urinary incontinence. Surface EMG data demonstrated significant test-retest reliability and significant clinical predictive validity for urinary stress and urge incontinence. Pelvic floor muscle sEMG is reliable and consistently predictive of several important clinical status variables, it can be a useful tool in early detection and prophylactic intervention for muscle laxity. Recent advances in sEMG technology make it cost-effective, convenient and easy to learn and administer by trained assisting staff. This technology is a powerful complementary tool for digital assessment of pelvic floor muscles and should be considered for use in gynecologic practice. Prenatal exercise programs, supported by pelvic floor muscle exercises, should be recommended for pregnant women, especially those who are accustomed to higher exercise intensity (179–181). Surface EMG using intravaginal probes is of widespread use as a biofeedback technique as well as for assessing pelvic floor muscles activity in women. Many muscles are involved and the issue of crosstalk during intravaginal sEMG recordings has been reviewed in Flury et al. (182). A gap in knowledge affecting sEMG investigation methods was identified by these authors. Literature addressing the proper electrode location and the crosstalk problem is scarce and often flawed. Conclusions are regularly drawn from an insufficient basis of evidence. Further research and training of operators is required (182).

High density surface EMG (HDsEMG) signals have been used for mapping the activity of the muscles surrounding the vaginal, the urethral and the anal canals (183, 184). Hacad et al. observed that continent and incontinent male patients presented sEMG changes during the first 6 months after radical prostatectomy that could be justified by the denervation/reinnervation of the external urethral sphincter (185).

#### Prenatal sEMG of the Anal Sphincter to Predict the Impact of Episiotomy

**Figure 7:**
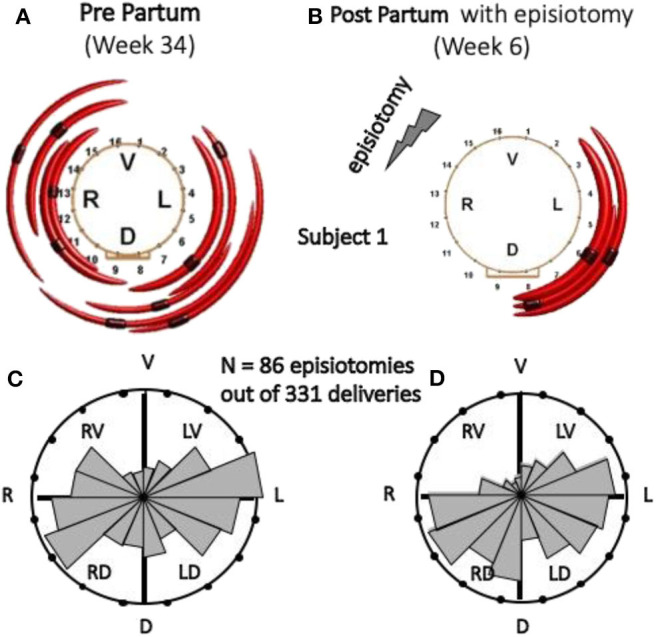
Effect of episiotomy on the EAS innervation pattern. The identified EAS motor units, indicated by red arcs, are not necessarily all the motor units of the EAS. **(A,B)** Identified MUs and their IZs, 4–6 weeks pre-delivery and 6 weeks post-delivery with right mediolateral episiotomy, in one subject. **(C,D)** Circular histograms of the number of EAS IZs pre- and post-delivery in 86 cases of right episiotomy (out of 331 deliveries). Both histograms are normalized with respect to the highest bin. The change in the RV quadrant of the EAS is statistically significant (123). V, ventral; L, left; D, dorsal; R, right. **(A,B)** Reproduced with permission from Cescon et al. (123), **(C,D)** Reproduced, with permission from Di Vella et al. (186).

Although very controversial and discouraged, episiotomy is still a widely performed surgery during child delivery. The techniques described above for the location of MU innervation zones (IZ) provide a tool to estimate the risk of partial denervation of the external anal sphincter (EAS) consequent to episiotomy. An intra-anal probe with a circumferential array of 16 electrodes detects the sEMG activity of the EAS during a voluntary contraction. Proper software identifies the location of the IZs of motor units of the EAS. This information can then be used, at the time of delivery, to guide episiotomy (if necessary) to the right or left side to minimize the risk of EAS partial denervation and possible future incontinence. Figures 7A,B show the MUs (and their IZs) detected in one subject at the 34th week of pregnancy and at the 6th week after vaginal delivery with right mediolateral episiotomy. Figures 7C,D show the distribution of IZs identified in 86 cases of episiotomy (all performed on the right side and out of 331 deliveries) around the electrodes of the probe. A statistically significant drop of the number of motor units innervated in the right-ventral (RV) quadrant of the EAS as well as the post-delivery reorganization of the EAS motor units are evident in Figures 7C,D (123, 125). This technique could be used as a biofeedback modality to retrain the muscle as is done with muscles surrounding the vaginal canal.

#### Applications in Ergonomics

**Figure 8:**
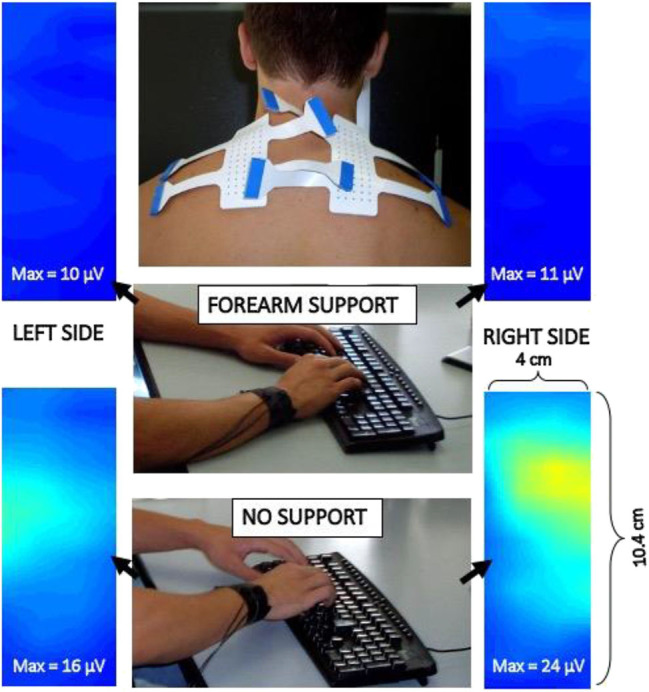
Example of two electrode grids applied to the trapezius muscle to study its activity during typing on a keyboard with and without arm rest on the desk. Images are interpolated and show the sEMG RMS distribution in space (see movies at URL https://www.robertomerletti.it/en/emg/material/videos/f6/ and https://www.robertomerletti.it/en/emg/material/videos/f7/).

Surface EMG techniques in ergonomics and occupational medicine for prevention and monitoring of occupational disorders were developed in the 90s (49, 187) and are currently applied for assessing chairs, posture, occupational tasks, fatigue, and risk at work (188–190). As an example, Figure 8 shows maps of sEMG RMS value (one 0.5 s epoch) of the trapezius muscle of a subject typing with and without forearm rest on the desk. Different activation levels of the upper part of the trapezius are evident while the subject is unaware of them. Teaching correct movements/efforts at work and prevention of work-related disorders are largely based on sEMG applications.

#### Applications in Exercise Physiology, Sports, and Aging

The literature concerning sEMG applications in sports is very extensive and focused on physiology (191, 192) training, prevention of injury, and recovery after injury (in particular the anterior cruciate ligament injury (193). Many sports have been investigated, in particular golf (194), jumping (195), cycling (196), sprinting (197), volleyball (198), but also strength training (199, 200), back pain in rowers (201), patellofemoral pain (170, 202), and aging (59, 203). The distribution of muscle fiber conduction velocity, related to fiber diameter, may provide insight in muscle structure. Mean/median spectral frequencies are affected by too many confounding factors to be used for this purpose but may be useful, in strictly controlled experiments, to monitor muscle fatigue resistance.

#### Applications in Gnathology

Temporomandibular disorder (TMD) is a term indicating musculoskeletal disorders in the jaw muscles and/or the temporomandibular joint. Surface EMG analysis has been employed to obtain a better understanding of TMD and is useful to elucidate the masticatory muscle function and adaptation in patients with TMD, using indices of dominance, asymmetry, co-ordination and co-operation of temporalis and masseter muscles during mastication. An association exists linking decreased activity to increased severity and asymmetry between affected and non-affected side in unilateral TMD patients (204–208). The ease-of-use of the sEMG assessment of masticatory muscles during static contractions and/or during chewing, has led to the development of standardized examination protocols and output graphs, which are suited for use in clinical routine assessment.

#### Other Applications

The above list of sEMG application fields is far from comprehensive. Many other fields take advantage of sEMG as a tool for either investigation, clinical assessment or treatment. These fields deal with the joint use of sEMG and ultrasound (209), the study of muscle deterioration in real or simulated (bed rest) microgravity conditions and the assessment of countermeasures (210, 211), the non-invasive detection of fasciculations (212, 213), the study of cramps (56, 214–216), the use of sEMG in rehabilitation games (217, 218).

## Barriers to Widespread Clinical Use of sEMG in Neurorehabilitation

### Current Situation

Section Surface EMG Applications provided a partial overview of the rapidly growing applications of sEMG in neuro-rehabilitation, movement sciences, occupational and sport medicine, and other fields. Many of the thousands of articles in these fields come from rehabilitation engineering groups, quite a few from movement science laboratories, but relatively few from the clinical world. Despite the extremely large number of publications about signal detection, processing tools, and small clinical studies, publications on routine applications in clinical practice are minimal. In addition, sEMG applications in medium/large clinical studies are rare (22, 64, 67, 73, 219). There is a lack of clinical studies (e.g., observational studies) verifying whether patients exposed to an additional sEMG assessment reach better outcomes than patients undergoing standard assessments do.

Hardware and software for sEMG detection and processing/interpretation has been developed mostly by academic researchers whose objective is to develop scientific innovation, and to publish their findings in respected science journals. In these cases, the research questions are typically technical, rather than clinical. The giant step between publishing a method, or developing a prototype, and the design, manufacturing, and marketing of a device for clinical applications is expensive and can be undertaken by companies only if there is evidence of clinical effectiveness and demand from the market. But a demand from the market implies some awareness of the users and their understanding of the potential use and relevance of the new method/device. If the users do not know why measuring muscle activity may be important, they will not be interested in a device or method to measure it. This brings up the issue of information, education and knowledge-transfer, but also other considerations concerning the potential consequences that making such measurement will imply. For example, who is qualified to decide on the measurement, who performs it, and who will use and interpret the resulting information to make decisions? Which balances will be affected, what will the costs or savings be, and who will gain something from doing it? This is important in private health management systems, where the role of insurance companies conditions the market, as well as in national health systems, where the state (that is the community) covers health cost with tax money.

Most of the scientific breakthroughs produced in academia do not routinely result in a marketable product or procedure. Commercialization of emerging technological innovations is difficult to accomplish; Transferring technological knowledge can also be a time-consuming process resulting often in a market failure. This may be because users are not yet competent or did not contribute to the development of the knowledge. On the other hand, academia-based researchers may also not be competent to assess the needs and boundaries of the clinical procedures. This scenario has been conceptualized as a “valley of death” between research and market. To tackle this general problem, major challenges are being analyzed, at the EU and national levels, and in the USA, involving educated/informed Communities of Practitioners (CoPs) in the process of effective transfer of high-value emerging technologies (220).

In addition to this, recent EU grants required the participation of companies as partners of funded projects.

### Some Fundamental Questions

To address the barriers to clinical use of sEMG, we believe it is important, together with the CoPs, to try to answer fundamental primary questions and a set of secondary questions which outline the potential added value that can be provided by sEMG-based assessments. Primary questions concern the pathophysiological status of the neuromuscular system as well as the definition/measurement of key characteristic features (e.g., Is the muscle on or off? Does muscle fatigue occur during a task? Is the control strategy changing during a task?). Secondary questions concern when and why the answer to a primary question is clinically important, who should answer the question and how this person should be trained. The list below includes a few examples of the many primary questions that can be answered by a competent analysis of sEMG:

1. Is the muscle active or not at a given time? When does the muscle turn on and off during a task?
2. Is the muscle relaxed or active or progressively changing its activation level? What level of force is produced?
3. Is muscle activity triggered by muscle lengthening and/or by the velocity of the stretch?
4. What is the level of muscle activation? Is the estimation of force (or force change) of any interest?
5. How are many muscles coordinated and what are the temporal relations between their activations?
6. Is there co-activation of different muscles during a task?
7. Is there a region of a muscle that is more or less active then other regions? Or is there a muscle of a group that is more or less active than other muscles of the same group?
8. What is the strategy adopted for controlling motor unit recruitment and muscle coordination?
9. Where are the innervations zones of the MUs of a muscle located along the muscle?
10. How long are the muscle fibers and how much are they shortening/lengthening during a task?
11. Is conduction velocity of the muscle fibers relevant for the situation at hand? What is its average value? What is its distribution across motor units normal or not?
12. Is muscle fatigue of interest in the situation at hand?
13. Is the number of active motor units of a muscle stable, or changing in time during a task? Are the active motor units rotating, that is are they being de-recruited and replaced by others during performance of a task?
14. What is the fiber size and fatigability of MUs?

The list below includes examples of secondary questions that should be asked and answered in association to each of the primary questions listed above:

1. When and why is the answer to this primary question important?
2. What must be measured to answer the primary question? What instrument should be used?
3. What is the knowledge required to answer this primary question?
4. Who is the competent clinical operator who should perform the measurements required to answer this primary question?
5. What is the knowledge that such operator must have in order to perform the measurement? How can this knowledge be acquired by this operator?
6. Who is the competent clinical operator qualified to interpret the results of the measurement and draw conclusions from them? How can the required competence and expertise be acquired?

The current limited clinical application of sEMG indicates that either type of questions are rarely asked. However, the literature unquestionably indicates that the answers to the primary questions are available and that they are important for understanding the pathophysiology of muscle conditions and motor control strategies. The issue is whether such questions are considered to be relevant, from the clinical viewpoint, by the CoPs, and which barriers prevent answering the relevant ones.

As indicated above and in section Introduction, large studies, and translational efforts from scientific knowledge to clinical application are hindered by a number of barriers a few of which have been previously investigated by other authors and identified as (a) lack of time, (b) lack of skills, (c) misperceptions of EBP (31, 37–40, 221–223). But these barriers go much beyond these issues and much beyond sEMG. They can be roughly grouped in four categories: cultural, educational, technical, and economical.

### Cultural Barriers

The cultural barriers limiting the widespread use of sEMG are not specific to this field and are the same that affect many other rehabilitation fields. They are, in general, related to the global approach to measurement in rehabilitation and to the concept of evidence provided by such measurements (39).

#### The Concepts of Measure and Measurement

In physics, “measurement” is the process of attributing a value to a physical quantity by comparing it to a standard reference quantity called “unit of measurement.” In rehabilitation, “measures” can be measurements of physical quantities (e.g., range of motion, 6-min walking test, etc.), ratings of a specific ability based on an ordinal scale with known levels (e.g., Functional Ambulatory Classification for assessing ambulation, etc.), ratings of multidimensional abilities on item scores that are summed up to obtain a total score (e.g., Barthel Index for assessing independence in the activity of daily life), results of questionnaires or aggregations of tests (pass/fail) or ordinal grades, such as very-poor/poor/sufficient/good/very-good, or 0/1/2/3/4/5 (e.g., for assessing force or resistance to stretch). The exact definition of each level of the scale may change from assessor to assessor. On the one hand, all these tools are useful, easy to be administered and represent a key element for both clinical activities and administrative procedures (e.g., reimbursements). Noteworthy, dichotomous variables are the pillar of epidemiological studies (e.g., exposed/non-exposed vs. dead/alive). On the other hand, they may suffer from metrical issues, from construct validity to sensitivity or reliability, and may lead to huge data-analysis problems when ordinal scores (e.g., 1/2/3/4/5) are treated as numbers and averaged or analyzed with parametric statistics and when the effects of a treatment are computed as numerical difference between scores obtained before and after the treatment. Moreover, electrophysiological variables, such as those describing motor control, cannot be assessed by clinical scales and require instrumental measurements. Some well-known textbooks, such as “Measurement in Neurological Rehabilitation” (224) do not even mention measurement of force/torque, or angular velocity, or sEMG and present only scales and questionnaires.

While physical measurements are the foundation of science and associate the change of a quantity as an effect due to some cause, the classification of the patient's current status or functional ability is the main goal of the “measures” in rehabilitation. Surface EMG amplitude (RMS, ARV) and spectral (MNF, MDF) features or timing during movement or tasks, are measurements of muscles signals that in turn reflect and quantitatively describe pathophysiological events or conditions or recovery level. In some cases, the possibility of turning this numerical information into clinically meaningful categories, based on a-priori knowledge and thresholds, would probably support the use of sEMG based examinations (but not of sEMG itself) in the rehabilitation practice.

#### Technology and Humanity: Communication Gaps and Lack of a Common Language

It has been properly pointed out that the statement “there can be no evidence in rehabilitation” that is so often heard from medical operators who think that their job is more “humanistic” than “scientific,” challenges the scientific basis of rehabilitation (3). Such thinking would drive physiotherapy and rehabilitation out of the mainstream of science. Rigorous reasoning and measurement-based approaches require a deep understanding of the physiological mechanisms and quantities being measured, of the instruments being used, and of the design of clinical studies in rehabilitation. Tradition and empirical experience alone are bad teachers (3).

Fundamental concepts of mathematics and biomechanics are associated to sEMG measurements and to the need for a language common to clinicians and rehabilitation engineers. Efforts in this direction are under way (e.g., Tutorials and CEDE consensus papers published in the Journal of Electromyography and Kinesiology) (52, 53, 61, 62). Applications of biomechanics and greater interactions between clinicians and rehabilitation engineers cannot take place without a common language that includes the concept of measurement of physical quantities with proper instruments (31, 38, 40). Rehabilitation medicine and physiotherapy are deeply connected with mathematics, physics and biomechanics. The fact that robotics and advanced technologies are entering the rehabilitation field (4, 6–8) is fear-inducing to some clinicians who consider these advances as job-threatening. Medical students and PTs should be taught to see technology as a tool in their hands. Rehabilitation operators demand more “intelligent,” fool-proof, and error-correcting devices to rely on (see section Technical Barriers). For this and other reasons, artificial intelligence, intelligent human-machine interfaces, and self-correcting data acquisition systems, are very important in rehabilitation and must be part of the training of professionals who should use them with proper competence and caution, and never totally rely on them.

#### Misunderstanding the Purpose of sEMG

Among rehabilitation professionals, there is a tendency to consider sEMG as a therapeutic tool so that the potential benefits of sEMG appear limited to biofeedback applications. In fact, sEMG is much more a monitoring tool, and occasionally, a diagnostic tool. The incorrect view of sEMG as a “therapy” is a barrier to its use.

### Educational Barriers

The clinical interpretation of sEMG is based on the timing, amplitude and the morphology (continuous activity, burst-like activity, MUAP shape and firing pattern, etc.) of the signal. Technical aspects related to the type of electrodes, the type of protocol used, the adopted filters, etc., affect the waveform, timing, amplitude, and spectrum of the signal. Also, the modification of the peripheral properties of the muscle and the modification of the central drive have an effect on the morphology of the sEMG signal (225). Although, unlike ECG and EEG, the wrong reading or interpretation of the sEMG tracing may not have dramatic consequences on the patient, it can change therapeutic decisions, surgical options, focal treatment of spasticity, and cost of therapy.

Reading a sEMG recording and linking a pathophysiological and/or biomechanical meaning to its features (that often result from computer processing) requires considerable competences. These are rarely available in the clinical environment. Educational barriers are a bottleneck. Many countries offer a Master in Health Professions (some specifically in physiotherapy). These degrees too often focus on legal, professional and administrative issues and neglect scientific and technical education. Noteworthy, The World Confederation for Physical Therapy (WCPT) advocates that the scope of physical therapist practice is not limited to direct patient/client care, but also includes research (https://www.wcpt.org/policy/ps-descriptionPT). The academic programs in movement sciences often provide a more scientific and research oriented background.

Only a few countries offer a Ph.D. program in physiotherapy and a few more offer a Ph.D. in movement sciences. Where there is no Ph.D., no research fellowships and research positions are available. This precludes the academic career of physiotherapists and has a profound impact on education. A 3-years (or 4-years, as in Belgium and The Netherlands, among other countries) BS program is barely sufficient to train a practitioner, not a contract professor or a clinical researcher able to promote and conduct large-scale studies. In addition, it is unthinkable that a practitioner will acquire this knowledge on his/her own time, in parallel to a heavy burden of clinical work, and publish in qualified journals to achieve an academic status (42). The sEMG field is deeply affected by this situation because of the need for clinical studies that can be carried out only by qualified researchers at the post-graduate level. Moreover, the lack of specific education also prevents the preparation of clinical application guidelines that must become a part of the education of all operators potentially involved in sEMG application.

In countries in which physiotherapy is not a graduate level degree, students are trained to become practitioners rather than clinical researchers. The concept of measuring physical quantities is neglected as well as the fundamentals of physics and biomechanics (from the physical point of view). Moreover, in countries that do not grant a PhD in physiotherapy or movement sciences, teachers of physiotherapy have in general, no or very limited research exposure or international experience. Insufficient continuing education and involvement of teachers in research projects is a barrier to clinical use of all new technologies, and sEMG in particular.

### Technical Barriers

Technological evolution led to the development of sEMG hardware that is simple to use and is commercially available. Powerful software can extract sEMG features whose clinical relevance is documented in the available literature. Nevertheless, there is a persistent demand for engineers to build systems that can be easily applied without a high risk of error. Users demand to be technically supported in the interpretation of signals and warned of potential misuse and acquisition error. There is a high demand for artificial expert systems and explanatory components that should be integrated into the sEMG systems and protect the user from errors and misinterpretations. However, no software will correct basic human errors (e.g., electrode misplacement, use of wrong filtering, etc.). This brings up the problem of the degree to which lack of competence can or should be replaced by expert systems, artificial intelligence or automatic devices. This may be a dangerous avenue of research in a field where developers and users have widely different expertise, experience and responsibility. Even if software is subject to the same stringent and reliable regulations as all medical devices, it cannot be fool-proof and cannot replace human expertise and competence. The solution is a more competent operator possibly assisted by a more intelligent machine providing warnings or “suggesting” possible interpretations.

Many researchers made remarkable efforts to (a) introduce sEMG as a tool to integrate biomechanical information for movement analysis, and (b) to provide tutorials and guidelines to clinical operators (29, 61, 108, 226–228). Very important contributions came from the European Project “Surface Electromyography for Non-Invasive Assessment of Muscles (SENIAM)” (226). Additional efforts are under way with the publication of a set of tutorials and consensus papers on the Journal of Electromyography and Kinesiology. These efforts have been designed to increase the competence of sEMG users, but their impact has been limited, suggesting that this may be a necessary but not sufficient step (229).

### Economic/Administrative Barriers

The new Medical Device Regulation (MDR) of the European Union requires proof of benefit through clinical studies, based on the Health Technology Assessment (HTA) procedure. This will be difficult to provide for sEMG equipment because of the lack of suitable personnel, creating a vicious circle. In addition, the producers of medical devices will have to maintain competencies in the areas of quality assurance and risk management. This is difficult to ensure, especially for very small companies, and may further hinder the translation of innovative sEMG procedures in the long term. Unresolved reimbursement issues and new regulatory barriers will hinder the development of sEMG systems adapted to clinical needs and their translation into clinical practice.

As mentioned by Duncan and Murray (221) “Whilst the importance of routinely measuring outcomes within the allied health professions is well-recognized, it has largely failed to be delivered in practice. Factors that influence clinicians' ability and desire to undertake routine outcome measurement are bi-directional: they can act as either facilitators or barriers. Routine outcome measurement may only be deliverable if appropriate action is taken at individual therapist, team, and organizational levels of an organization.” The European MDR might be such action.

#### Should sEMG Measurements Be Fast, Simple, Automatic, and Inexpensive?

In most countries, a physiotherapy session lasts 30–60 min and is usually related to treatment, not to measurement of results. This time constraint is mentioned in many articles reporting results of questionnaires or interviews to physiotherapists (38, 40). In their recent work, Feldner et al. (31) reported that “most clinicians (19 out of 22) relied primarily on clinical observation of functional skills … used palpation, manual muscle testing … their choices were often based on time constraints and reimbursement considerations.” These authors further noticed that “most clinicians (18 out of 22) reported that they received very little training specific to the use of sEMG systems during their professional curricula…” and “…perceived barriers in “convincing” department administration to invest in technology….”

In addition, these authors indicated that “Despite barriers, participants were eager to learn about sEMG, noting that it would not replace but enhance their current clinical methods….” Due to the lack of training, the request is that sEMG equipment and testing should be easy to self-learn, fast and simple to use and inexpensive. The lack of teaching associated to self-teaching by trial and errors or from salespersons only, causes user frustration and is a major barrier to the use of sEMG. This is not so in the case of ECG and EEG (and needle EMG) whose users are provided with proper academic education and training.

Cost of FDA or CE-approved sEMG equipment ranges from about 10 k$ to nearly 40 k$ for wireless systems providing up to 32 channels and processing software. This is in the same order of magnitude of inexpensive to sophisticated ECG, EEG, and needle EMG equipment.

A possible explanation for the differences between ECG, EEG, needle EMG, and sEMG is that the former have a higher diagnostic yield while sEMG only provides information on the functional level, which is associated with prevention, monitoring, assessment, and treatment planning but less to diagnosis. The lower importance attributed to the latter functions with respect to diagnosis is a bias that is hard to overcome and has high social and economic costs.

#### Coverage by Insurances and National Health Systems

A vicious circle exists between the need to collect more evidence of sEMG effectiveness in assessing results, and the lack of qualified clinical researchers able to do it. This is a clinical activity that should not be left to either the manufactures or to the rehabilitation engineers. It is a clinical activity dealing with, and requiring, studies on patients. Despite the large number of publications on small studies, the evidence does not seem to be sufficient to convince insurance companies or National Health Systems to reimburse the cost of sEMG-based testing for effectiveness of treatments. In North America, sEMG procedures are not routinely reimbursed by insurance, unless they are part of a preoperative protocol, such as used for surgical planning in patients with CP. This is in contrast to diagnostic procedures using intramuscular needles which are done routinely by clinicians for diagnostic reasons.

#### Research Funding

Evidence supporting the use of sEMG is only partially available because it is limited to small studies. Large studies require substantial funding and competent operators. Competent operators are lacking because of educational barriers. Educational barriers are lower in countries where post-graduate academic degrees are available. Therefore, large-scale studies should be proposed where researchers are available to implement them. Research funding is required to support researchers and pay for equipment and management of large studies.

## Conclusions

Hundreds of publications on peer-reviewed journals (Figure 1) provide a consistent body of evidence that the applications of sEMG makes appropriate information available in many medical fields, including the neurorehabilitation and orthopedic areas. Despite these achievements, clinical applications in health delivery institutions remain very limited because of many barriers. Clinicians have ready access to articles, evidence summaries, systematic reviews, and meta-analyses that assess and summarize the state of the art in the field. However, scientific publications are necessary but not sufficient to promote innovation. As indicated in a well-known editorial by A. Jette in 2017 (229) “Publishing our work in journals is essential—but publication of research is not, by itself, sufficient if our goal is to change clinical practice. People follow the lead of other people they know and trust when they decide whether to take up an innovation and change the way they practice!”

In this work, the barriers to a widespread use of sEMG have been classified into four main groups: cultural, educational, technical, and economic. These are strictly linked and interdependent.

Cultural barriers derive from “uneasiness” with technology, from communication gaps, different perceptions and approaches between rehabilitation engineers and clinical operators. These different perceptions hinder technology transfer and generate educational barriers. Overcoming these barriers requires a strongly interdisciplinary educational approach. The lack of a partially overlapping high-level education, involving rehabilitation professionals and engineers, results in different languages, communication gaps, different approaches to common problems, or, in one word, cultural barriers that delay technology transfer. The development of common languages at common institutions would promote the use of sEMG systems and other measurement techniques

To this end it is important to point out and underline the series of open access Tutorials (61, 62) and of Consensus Papers within the “Consensus for experimental design in electromyography (CEDE)” project (53) promoted by the Journal of Electromyography and Kinesiology.

Overcoming educational barriers requires (a) a greater degree of bidirectional osmosis between the clinical and the research environments, (b) funding of translational efforts, (c) use of textbooks and manuals related to the clinical use of sEMG in specific applications prepared by experts (19), (d) design and implementation of large clinical studies. These should rise from (a) simple case-series on the added value provided by sEMG assessment aimed to the selection of a proper treatment, (b) observational studies comparing both pathways and outcomes of cohorts of patients treated in centers with/without sEMG adjunctive assessments, up to RCTs addressing the percentage of modified treatments and the differences in the functional outcomes determined by the use of sEMG-based adjunctive assessments. These activities must be carried out by qualified researchers within post-graduate research programs. This brings up the need for new academic figures merging clinical and physiopathological competences with the capability of understanding and properly using state-of-the art sEMG instrumentation/technology.

The lack of higher academic degrees in physiotherapy and movement sciences prevents (a) the education of qualified researchers able to properly apply the rapidly developing technology and to carry out large clinical studies, and (b) continuing education initiatives in teaching and research to exploit the growing assessment capabilities provided by technology.

Technical barriers are due to (a) sEMG systems considered unfriendly, (b) the lack of familiarity with hardware and signal processing/interpretation techniques, and (c) the demand for fool-proof automatic equipment. The demand for support in the interpretation of signals and automatic warning of potential misuse and acquisition errors cannot be fully satisfied. Automatic expert system are no substitutes for human expertise and competence and may be misleading. No device should be used without knowledge of its performance, limitations and misuse, and without user's critical competence. Education and research should be institutionally planned and provided, like in other fields, at the academic level, by training new figures with a strongly inter- and multi-disciplinary approach. They will, in turn, train a new breed of clinical operators able to manage technology and interact with engineers and manufacturers.

Finally, economical barriers, including cost/benefit analysis, should be seriously considered to identify the most economically rewarding sEMG-based applications, thus turning boundaries into project specifications. This requires fellowships for training researchers and funding for support of large clinical studies whose results will lead to reduction of the economic burden of institutions paying for treatment costs.

## Data Availability Statement

The original contributions presented in the study are included in the article, further inquiries can be directed to the corresponding author/s.

## Author Contributions

All authors listed have made a substantial, direct and intellectual contribution to the work, and approved it for publication.

## Conflict of Interest

The authors declare that the research was conducted in the absence of any commercial or financial relationships that could be construed as a potential conflict of interest.
